# P043. Hyperechogenicity of the periaqueductal gray in chronic migraine and episodic migraine as a potential marker of progressive dysfunction: preliminary results with transcranial sonography

**DOI:** 10.1186/1129-2377-16-S1-A61

**Published:** 2015-09-28

**Authors:** Elena Guaschino, Natascia Ghiotto, Cristina Tassorelli, Vito Bitetto, Giuseppe Nappi, Arrigo Moglia, Daniele Bosone, Grazia Sances

**Affiliations:** Clinical Neurophysiology Unit, University of Pavia, Pavia, Italy; Headache Science Centre, National Neurological Institute C. Mondino, Pavia, Italy; Department of Brain and Behavioural Sciences, University of Pavia, Pavia, Italy

## Background

It is widely accepted that the brainstem plays a role in migraine pathophysiology. The periaqueductal gray matter (PAG) is a substantial component of the descending pain modulatory network. Previous MRI studies by Welch et al demonstrated that PAG iron levels are abnormally high both in episodic and chronic migraine, suggesting the hypothesis that iron accumulation may be a marker of progressive PAG dysfunction. The increase in iron levels can be investigated with transcranial sonography (TCS) because of heavy metal-induced hyperechogenicity.

## Aim

The purpose of our study was to evaluate hyperechogenicity of PAG in patients with episodic migraine (EM) and in subjects with chronic migraine associated with medication-overuse headache (CM+MOH).

## Methods

We evaluated 10 patients diagnosed with EM and 10 patients with CM+MOH: one patient from group 1 and one from group 2 were excluded because of unsonable transtemporal window. TCS was performed using Acuson Sequoia ultrasound machine with a 2-MHz transducer. The sonographic parameters were set according to standard literature criteria. Using the transtemporal approach, the midbrain and diencephalic examination planes were visualized in axial section. Echogenicities of raphe midbrain, substantia nigra and PAG, thalami, lentiform nucleus and head of the caudate nucleus were examined and graded as hyperechogenic. The maximal width of the frontal horns of the side ventricles and the minimal transverse diameter of the third ventricle were measured on a standardized diencephalic examination plane. Hyperechogenicity was considered as the visually rated intensity of the ultrasound signal increase compared to the surrounding brain tissue.

## Results

PAG echogenicity was higher in patients with CM+MOH than in those with EM: 6/9 CM+MOH patients showed PAG hyperechogenicity, while only 1/9 in the EM group.

## Conclusions

These preliminary findings suggest the occurrence of a progressive degeneration of PAG in migraine. The possibility to reliably detect it with TCS would provide a low-cost, harmless, widely available tool. Evaluation on a larger population is needed to confirm PAG dysfunction in chronic migraine and the reliability of TCS for screening purpose and to predict the evolution of the disease.

Written informed consent to publication was obtained from the patient(s).

## Conflict of interest

None.

